# Valuing the scholarship of integration and the scholarship of application in the academy for health sciences scholars: recommended methods

**DOI:** 10.1186/1478-4505-5-5

**Published:** 2007-05-29

**Authors:** Anne Hofmeyer, Mandi Newton, Cathie Scott

**Affiliations:** 1 Faculty of Nursing, University of Alberta 3^rd ^Floor, Clinical Sciences Building, Edmonton, Alberta, T6G 2G3, Canada; 2 Knowledge Utilization Studies Program, 5-112 Clinical Sciences Building, University of Alberta, Edmonton, Alberta, T6G 2G3, Canada; 3 Knowledge into Action Department, Calgary Health Region, 10101 Southport Road, Calgary, Alberta, T2W 3N2, Canada; 4 Department of Community Health Sciences, Faculty of Medicine, University of Calgary, 3330 Hospital Drive NW Calgary, T2N 4N1, Canada

## Abstract

In the landmark 1990 publication *Scholarship Reconsidered*, Boyer challenged the 'teaching verses research debates' by advocating for the scholarship of discovery, teaching, integration, and application. The scholarship of discovery considers publications and research as the yardstick in the merit, promotion and tenure system the world over. But this narrow view of scholarship does not fully support the obligations of universities to serve global societies and to improve health and health equity. Mechanisms to report the scholarship of teaching have been developed and adopted by some universities. In this article, we contribute to the less developed areas of scholarship, i.e. integration and application. We firstly situate the scholarship of discovery, teaching, integration and application within the interprofessional and knowledge exchange debates. Second, we propose a means for health science scholars to report the process and outcomes of the scholarship of integration and application with other disciplines, decision-makers and communities. We conclude with recommendations for structural and process change in faculty merit, tenure, and promotion systems so that health science scholars with varied academic portfolios are valued and many forms of academic scholarship are sustained. It is vital academic institutions remain relevant in an era when the production of knowledge is increasingly recognized as a social collaborative activity.

## Background

Over 25 years ago, Mensah [1] identified many *dysfunctional manifestations *in the process of tenure and promotion that result from judging an academic scholar's ability and worthiness using a yardstick that measures only the success rate of research grant applications and the quota of publication outputs. She noted there was little value placed on one's success in classroom teaching or on service activities that made a difference outside the academic community but that directly flowed from academic work. Further, she claimed that the pressure to publish had influenced a rise in 'intellectual dishonesty, academic espionage, academic plunder, the me-orientated attitude, disinterest in students and teaching, as well as a lack in collegiality' [1]. She argued that links between the ethic of publish or perish and market forces had shifted the focus from the major responsibility of teaching. More recently, Ernest Boyer [2] reported that more than half the faculty working in academic institutions believed the pressure to publish diminished teaching quality. Faculty reported feeling torn between competing demands of research work and teaching courses and felt compelled to 'take short cuts in their research or rely heavily on teaching assistants – an arrangement that is often less than satisfactory for both student and professor' [2].

In the landmark 1990 research report, *Scholarship Reconsidered*, Boyer reconfigured academic scholarship into four dimensions: discovery, teaching, integration, and application. Boyer advocated a return to the broader view of scholarship but stressed the four dimensions should be evaluated as separate yet overlapping areas [2]. Glassick and colleagues continued the work in the area of evaluation and quality measurement in the 1997 publication, *Scholarship Assessed *[3]. While considerable work has been undertaken to define the scholarship of teaching and the methods to document its value (see for example: [4,5]), sustained change in emphasis within the health sciences academy is a contested and complex process.

In this article, we contribute to the less developed areas of the scholarship of integration and the scholarship of application in the health sciences academy. We consider Boyer's theory of scholarship for health sciences scholars and an approach to evaluate the scholarship of integration and application using the six standards developed by Glassick and colleagues [3]. We discuss a participative framework to guide institutional change and propose a method to report and evaluate the scholarship of integration and application as part of our recommendations. We conclude with a call for structural and process change in the merit, tenure and promotion policies to value all health science scholars' varied forms of academic scholarship; this change must include equitable evaluation strategies.

## Creating a sense of urgency for participation in the health sciences

There is increasing interest in the challenges and mechanisms related to reporting non-traditional academic scholarship productivity and contributions to applied scholarship for health services research [6,7]. Today's academy is stressed by reduced operating budgets which have influenced financial planning decisions by university administrators. This fiscal climate has resulted in rigid rules in terms of career advancement, tenure and promotion that directly link to scholars' performance in research and publishing records [1,8] while teaching and service are disproportionately undervalued [9]. In 2000, Gros Louis asserted that academic institutions still maintain a traditional approach in what is counted in reward, promotion and tenure systems that remains puzzling to those outside its walls and remains contentious to those within the system. Questions commonly asked about this issue include: "How much research is enough? How good does it have to be? Does it have to be funded, at what level, and by whom? How widely cited and read? How do we know if someone is or is not an excellent teacher? What counts and what does not count as service to the profession and to the university?" [10]. Asking the questions about research, teaching and service is a start, but obtaining unambiguous answers and linking these answers to rewarding the scholarship of discovery, teaching, integration and application in the academy is the more difficult work.

The current view of scholarship as accrued research grant dollars and publications (the scholarship of discovery) runs counter to academic institutions' obligations to serve society and improve the health and wellbeing of communities using applied and integrated scholarship. In this sense, discovery scholarship fails to reflect increasing awareness of the contextualized nature of knowledge production [11]. This is especially evident in the health sciences academy. Increasingly blurred lines between science and society require that we re-think how academic contributions are valued both within and beyond the academy. Publishing in journals with a high impact factor is viewed as a measure of quality, but most certainly does not guarantee dissemination of scholarship activities to community stakeholders [12,13]. New rules of the game should strike a balance between the current demands to publish or perish and the newly required emergent call for the academy to 'participate or perish' [11]. Recognizing and weighting applied scholarly activities for tenure and promotion involves conversations between researchers and administrators in faculties and departments in the academy [6].

## Health science scholars and interrelated areas of scholarship

In academic institutions, scholars most often pursue the scholarship of discovery with specific emphasis on thinking and developing knowledge with other scholars [14,15]. Boyer [2] proposed that a scholar works in the four interrelated areas of scholarship in the pursuit of knowledge that is responsive to human problems and societal needs. This broader perspective of interdependence between academic institutions and society in the application of new knowledge to address societal problems makes the case for the scholarship of application and integration. Academic institutions also benefit because there is potential to fulfill their societal obligations through the interrelated areas scholarship [16].

### The scholarship of discovery

The scholarship of discovery is understood as original research that expands or challenges current knowledge in a discipline. Boyer [2] defined discovery as the creation of knowledge for knowledge sake; its purpose is to contribute not only to knowledge but also to the intellectual climate of academic institutions. Questions asked by scholars of discovery include: What should be known?; and, What has yet to be found? [2]. New knowledge is vetted and regulated through peer-evaluation via publications. While this commodity is most important in the merit, promotion and tenure reward systems in the academic institutions, this traditional view of scholarship marginalizes other forms of scholarship and is a powerful disincentive to those who are pursuing tenure and promotion but who are more active in teaching, integration, and applied scholarship.

### The scholarship of integration

The scholarship of integration is closely related to the interprofessional debates; it relates to making connections across disciplines and shaping a more coherent and integrated use of knowledge. Integration work is creative connectedness, interpretation and synthesis, so is closely related to discovery, but poses somewhat different questions in terms of meaning and impact. This form of scholarship interprets meaning to isolated facts and creates new perspectives that can answer questions not originally possible to answer. Health science scholars engaged in integration require innovative thinking to be able to integrate knowledge from different disciplines and create new and different perspectives on significant ideas and theories [17]. Such scholars ask questions that require critical analysis and interpretation such as questioning what the research findings mean and whether it is possible to interpret what has been discovered in ways that provide a larger, more comprehensive understanding [2].

Previously located on the margins of academic endeavor, the scholarship of integration is now central because it is definitely best equipped to respond to contemporary problems at both an individual and societal level [2]. Researchers are locating their discovery work, or that of others, into broader intellectual patterns, thus moving beyond the disciplinary silos to build interdisciplinary partnerships with capacity to respond to multi-focal, complex human problems. Moreover, funding bodies are increasingly supportive of collaborative, integrated partnerships and teams as a way to generate knowledge and new approaches.

### Scholarship of application

In application scholarship, health science scholars build bridges and collaborative relationships with other disciplines, decision and policy-makers and communities in order to apply theory to solve every-day problems. Application scholarship directly links other forms of scholarship with practice [14,17,18]. This process involves dynamic engagement and the translation of new knowledge in practical interventions that solve problems or improve the difficulties experienced by individuals and society [2,16]. Hall states this scholarly activity allows dynamic creativity, allows new public policies, allows theory and practice to renew each other and allows "the academic world to climb down from its ivory tower" [14]. Health science scholars engaged in applied scholarship seek to understand how knowledge can be responsibly and ethically applied to consequential problems and how it can be helpful at micro (individual), meso and macro levels (society, government, institutions), as well as seek to learn how social problems *themselves *can define an agenda for scholarly investigation [2].

### The scholarship of teaching

The scholarship of teaching must extend beyond simply transmitting information to a process that is also transforming and extending the learning of students and scholars [2]. In this sense, the scholarship of teaching involves stimulating active learning, critical thinking and the commitment to life-long learning. Recent debates have centred on how to differentiate between the scholarship of teaching and teaching excellence and the relationship of this scholarly pursuit to other forms of scholarship [19]. Moreover, considerable weight is now placed on student evaluations of teaching received which may reflect their personal satisfaction related to grades assigned to their work, rather than the merit of the teaching by the scholar and the curriculum.

## Evaluating the different areas of academic scholarship

Soon after *Scholarship Reconsidered *was published in 1990, questions arose about how to evaluate and reward Boyer's four forms of scholarship. What worked to assess the quality of discovery scholarship in the past could not meaningfully assess the new fields of scholarship [18]. Borne out of this critique was the development of six assessment standards for scholarly work: (1) clear goals; (2) adequate preparation; (3) use of appropriate methods; (4) achievement of significant results; (5) effective presentation and communication of results; and (6) reflective critique of one's work [3]. These six standards provide clear criteria for excellence and a framework for reporting the application and integration forms of scholarship as well as their combined contribution as 'community scholarship'. Further, these standards can be considered a springboard for Faculty in developing their own interpretations of scholarship [20].

While there is general agreement that the scholarship of discovery and teaching are relatively straightforward to evaluate, Calleson and colleagues [21] argue that applied and integrated scholarship are not as easily quantified. Glassick and colleagues [3] proposed specific questions to evaluate the six standards of scholarship. Their work was extended by Aday & Quill [22] who developed specific questions to evaluate integration and application scholarship. Maurana and colleagues [23] contributed questions to evaluate community scholarship and engagement. This literature is succinctly presented in Table 1.

**Table 1:** Evaluation of Integration, Application and Community Scholarship

**Six Standards to Evaluate Scholarship**^3^	**Integration**^22^	**Application**^22^	**Community scholarship**^23^
**1. Clear Goals**: • Does the scholar state the basic purpose of his or her work clearly? • Does the scholar define objectives that are realistic and achievable? • Does the scholar identify important questions in the field?	Framework for the integrative perspective is elaborated clearly	Policy or social problem being addressed is specified clearly	1. Are goals clearly stated and jointly defined by community and academics? 2. Has partnership developed goals, objectives based upon community needs? 3. How do we identify community issues? Are needs and issues recognized by scholar and institution? 4. Do both community and academia think issue is significant, important? 5. Have partners developed a definition of what "common good" is? 6. Have partners worked toward an agreed upon "common good"? 7. Is there a vision for the future of partnership?
**2. Adequate Preparation:** • Does the scholar show an understanding of existing scholarship in the field? • Does the scholar bring the necessary skills to his or her work? • Does the scholar bring together the resources necessary to move the project forward?	Approach evidenced breadth and depth in the topics being addressed	Approach evidences having laid the relevant groundwork with participating entities	1. Does the scholar have knowledge and skills to conduct assessment and implement program? 2. Has scholar laid groundwork for program based on recent work in the field? 3. Were needs and strengths of community identified and assessed using appropriate method? 4. Did the scholar and community consider all the important economic, social, cultural and political factors that affect the issue? 5. Does the scholar recognize and respect community expertise? 6. Have community-academic partners become a community of scholars? 7. Does scholar recognize community can 'teach' and has expertise? 8. Does scholar stay current in the field?
**3. Appropriate Methods:** • Does the scholar use methods appropriate to the goals? • Does the scholar apply effectively the method selected? • Does the scholar modify procedures in response to changing circumstances?	Author's perspectives (or biases) in the selection and synthesis of materials are articulated explicitly	Approach balances both rigor and relevance	1. Have all partners been actively involved at all levels of partnership process, assessment, planning, implementation, evaluation? 2. Has development of partnership's work followed a planned process that has been tested in multiple environments, and proven effective? 3. Have partnerships been developed according to a nationally acceptable framework for building partnerships? Approach 1. Are methods used appropriately matched to the need? 2. Do methods build community involvement and sustainability? 3. What outcomes have occurred in program development, implementation? 4. Do scholar and community select, adapt and modify method with attention to local circumstances and continuous feedback from community? 5. Do programs reflect culture of community? 6. Does scholar use innovative and original approaches?
**4. Significant Results:** • Does the scholar achieve the goals? • Does the scholar's work add consequently to the field? • Does the scholar's work open additional areas for further exploration?	Results influence or inform interdisciplinary perspective	Results influence or inform program or policy design	1. Has program resulted in positive health outcomes in community? 2. Has partnership effected positive change in community and academic institution? 3. Have models been developed that can be used by others? 4. What has been the impact on community? 5. What has been the impact on academic institution? 6. Have external resources (e.g. grant, fund-raising) been affected by program? 7. Are results effective as judged by both community and academia? 8. Do scholar and community commit to long-term partnership?
**5. Effective Presentation:** • Does the scholar use a suitable style and effective organization to present his or her work? • Does the scholar use appropriate forums for communicating work to its intended audiences? • Does the scholar present his or her work with clarity and integrity?	Results are presented clearly and interpretable to interdisciplinary audience	Results are presented clearly and interpretable to interdisciplinary audience	1. Has work (outcomes and process) of partnership been reviewed and disseminated in community and academic institutions/ 2. Have there been presentations and publications on community-based efforts at both community and academic levels? 3. Are results disseminated in a wide variety of formats to appropriate community and academic audiences?
**6. Reflective Critique:** • Does the scholar critically evaluate own work? • Does the scholar being appropriate breadth of evidence to his or her critique? • Does scholar evaluate to improve quality of future work	Limitations of one's own and other's research across disciplines are identified	Evidence for evaluating policy or program impact is available	1. What evaluation has occurred? 2. Does scholar constantly think and reflect about the activity? 3. Would community work with scholar again? 4. Would scholar work with community again?

Evaluation of the scholarship of integration in the health sciences academy can review whether academic publications written for 'nonspecialist' audiences demonstrate analytical and literary ability to translate complex scholarship into accessible, relevant messages. Evaluation can focus on whether this work demonstrates a clear understanding of the disciplines involved and whether key issues have been defined and creative insights presented. Further, how well the essential message has been delivered and how the non-specialist has responses to the work can be examined (e.g., in what ways has the public or other discourse been advanced?) [2]. In the context of academic institutions' obligations to improve the human experience and society, the legitimacy of this scholarly work is clearly obvious.

Evaluating the scholarship of application is predicated upon identifying examples of applied scholarly activities and developing comprehensive reporting methods. There must be a direct correlation between the intellectual work of the scholar and the applied work, such as consultation, evaluation and analysis [2]. Reporting applied scholarship should include the evaluation by the scholar, and by the recipients of the service, such as decision and policy-makers and communities. Those assessing applied scholarship should ask whether the activity is directly related to the academic expertise of the scholar and whether project goals have been defined, whether procedures have been well planned and whether actions have been carefully recorded. Further, evaluation should include how the endeavor has not only benefited the community, but also added to the scholar's own understanding of their health sciences expertise [2]. Calleson et al. [21] also recommend that 'applied products' and 'community dissemination products' be assessed as scholarship contributions alongside peer-reviewed articles. Valued by communities are applied products to improve health, such as programs, guidelines, policies, resource materials, technical assistance and training. Community dissemination products include media reports, websites, and presentations. A similar concept of community scholarship, engagement, and productivity was proposed by Maurana and colleagues [23] who suggested the work must be responsive to the needs and problems defined by the community and result in enhanced health. However, work with communities will only be defined as scholarship when there is clear evidence of links with current research findings and discipline specific knowledge, when the products are peer reviewed and are available for public scrutiny, use, evaluation [23].

Scholarly integration and application activities can be reported in a mechanism such as a Creative Professional Activity Dossier [6,24]. The dossier can work as a reflective document in which scholars draw links between theories and evidence, provide a description of their applied scholarly activities, the underlying guiding rationale/evidence, their objectives, the evidence of impact, and a range of supporting documentation [6].

Those evaluating the dossier could consider:

• the creativity and quality of the integration and application activities and the degree of impact on the profession, which is considered more important than the quantity of activities and achievements;

• whether the activities are current and able to be sustained;

• in what way the contributions and activities are relevant to the institutions; and

• whether there has been a resulting change in policy, organizational-decision making, or clinical practice [24].

## Recommendations supporting the broader research perspective

Academic work in the health sciences field has been traditionally categorized as research, teaching and service. Service is understood as doing good works in the university such as committee work and citizenship activities to benefit the community but it is important to distinguish between social and civic service (citizenship) and scholarship. As stated earlier, to be considered as applied scholarship, service activities must directly relate to health scholars' specific area of knowledge and professional expertise and exhibit intellectual rigor [2,17]. A more inclusive and integrated view of what it means to be a health sciences scholar is needed. This view must value new knowledge generated through research, teaching synthesis, and practice [2]. An open, social process of inquiry that represents a synthesis of academe and community influences would facilitate greater reflexivity of the complexities, risks and benefits [11].

The health sciences academy is well equipped and positioned to attend to the interests of the larger community and to serve as a bridge between academia and society by linking theory and practice through applied and integrated scholarship in addition to teaching and discovery scholarship. To prevent further disengagement from applied scholarship, academic institutions must take the necessary risk to legitimize and to equally reward all forms of scholarship for the benefit of scholars who engage in each area of scholarship [25,26]. Although much has been written about how traditional reward systems need to be restructured in order to recognize a broader definition of scholarship, it is unclear whether or not the professoriate in general will embrace their new interdisciplinary roles and address complex social issues in pursuit of their careers [25].

We strongly advocate for the following changes and elaborate more specifically on the final two recommendations:

• Health science faculties to embrace diversity and creativity in scholarship and to take any necessary risks involved in changing the status quo of privileging traditional scholarship, and those who generate it.

• Allowing scholars to focus on two or three areas of scholarship (i.e. discovery, teaching, integration and/or application) during an agreed period and evaluating the scholarly work over that agreed period of years.

• Support is unequivocally provided to scholars engaged in interdisciplinary partnerships within academic institutions and communities, and rewarded in merit, tenure and promotion systems.

• Naturally evolving communities of scholars are valued and mutually respected by colleagues.

• A framework to guide change be adopted for encouraging participation by all involved in the change.

• A method to compile evidence in a portfolio format that reports the scholarship of integration and application be widely adopted.

## A framework to guide change

The inconsistency between mission statements of academic institutions and tenure and reward structures is socializing health science scholars away from the scholarship of integration and application. This reality must change given the position of governments, policy makers and professional bodies that premises healthcare system renewal on scholars and community-based professions working together in interprofessional teams for societal benefit [27-29]. For this to occur, however, there needs to be greater congruence between the institutional mission statements that claim to value all four scholarship dimensions and the explicit policies about tenure-track appointment, incentives, evaluation, rewards, merit, promotion, and tenure in health science faculties. This process is best achieved by academies and their faculties reaching consensus about their own definitions of each dimension of scholarship and develop agreed examples in each area [30].

We suggest adopting a participative approach using Kotter's eight-step change framework to encourage participation by all involved in the change within and beyond the academy [31]. Step 1 is about developing a sense of urgency for change which we suggest is gathering momentum in the health sciences literature. Step 2 is about creating criteria to report and evaluate the scholarship of integration and application. We consider that Steps 3 to 8 proposed by Kotter [31] are best developed between scholars in the academy and with relevant stakeholders in the community. These steps encourage faculty to adopt a broad scholarship reporting mechanism, to leverage support from influential players, and to foster the cultural change that accompanies formal structural change in tenure and promotion systems.

### A Portfolio method

The Portfolio method is designed to explain the objectives of the scholarship of integration and/or application activities, the rationale thinking that underpinned each activity, and provide evidence of impact [6]. The compilation of evidence aims to address a range of evaluative questions (illustrated in Table 2). These evaluative questions serve to firstly guide the scholar in organizing relevant evidence and second, serve as a guide for the evaluator in the process of assessing the evidence. The process of developing the Portfolio contributes to scholarship through self-reflection, self-evaluation, and self-development. The Portfolio is a means to report on knowledge transfer and exchange activities and includes products produced in collaboration with others as supporting documentation. The Portfolio could be five to eight pages in length, plus supporting documentation (appendices) and organized in four sections: (1) philosophy and clear goals; (2) contributions to the scholarship of integration and application; (3) reflections and assessment; and (4) supporting documentation presented as appendices.

**Table 2:** Scholarship of Integration and Scholarship of Application Portfolio

**Scholarship of Integration and Scholarship of Application Portfolio**
The **scholarship of integration **gives meaning to specific discoveries by making connections within and between disciplines, locating knowledge in a broader context, making connections and synthesizing knowledge. The **scholarship of application **is focused on engagement with the broader community and the use that might be made of knowledge to address societal problems^1,2,3,7^
The Portfolio is designed to explain the objectives of the scholarship of integration and application activities, the thinking that underpinned it, and to demonstrate impact^1,3,6^. Developing the Portfolio contributes to scholarship through self-reflection, self-evaluation, and self-development. The Portfolio is a yearly record and a cumulative record of process and outcomes. Yearly records contribute to a cumulative record of historical data that will be useful when compiling award, grants, tenure and promotion applications^4^. Scholars include rationale for and description of each activity, and provide evidence of impact. The Portfolio reports knowledge transfer and exchange activities and products produced in collaboration with communities (engagement)^23^. It can be five to eight page document (plus appendices) organized into four sections:
1. Philosophy and clear goals
2. Contributions to the Scholarship of Integration and Application.
3. Reflections and Assessment.
4. Supporting Documentation^4^.
**1. Philosophy and clear goals**
1.1 Statement of your *philosophy *about the scholarship of integration and application, your *goals *and the theory, evidence and/or rationale guiding your work **(Appendix A)**
**2. Contributions to the Scholarship of Integration and Application**
2.1 *Scholarship Activities and Funding awards*: Objectives of projects and activities, describe underpinning thinking and theories, and how these activities contribute to mission of the academic institution and your discipline **(Appendix B)**
2.2 *Activities to Improve Scholarship: *Development and training to improve effectiveness **(Appendix C)**
2.3 *Committee Service regarding Scholarship and Scholarship Issues*: Faculty, academic institution and community activities to strengthen scholarship **(Appendix D)**
2.4 *Knowledge Exchange Activities*: Peer reviewed papers. Dissemination documents for wider audiences. Applied products to facilitate immediate transfer of knowledge into application. Plain language reports. Community dissemination products^2,23 ^**(Appendix E)**
**3. Reflection and Evaluation of Impact**
3.1 *Reflections on Scholarship impact*: Summary statements that reflect your assessment of the effectiveness, impact, and results of your scholarship **(Appendix F)**
3.2 *Integration scholarship*: non-academic publications that are written for non-specialist audiences that show a critical, analytical and literary ability to interpret and translate complex scholarship into accessible communication. Questions for evaluators of this scholarship include: What do the findings mean? Is it possible to interpret what's been discovered in ways that provide a larger, more comprehensive understanding? Does the work show a careful understanding of the discipline? Have the key issues been well defined and creative insights well presented? Has the essential message been clarified? In what ways has the public discourse been advanced?^2^
3.3 *Application scholarship *– questions for evaluators include: Is the activity directly related to the academic expertise of the professor? Have project goals been defined, procedures well planned and actions carefully recorded? In what ways has the work not only benefited the recipients of such activity, but also added to the professor's own understanding of their academic field? How can knowledge be responsibly applied to consequential problems? How can it be helpful to individuals as well as institutions? Can social problems themselves define and agenda for scholarly investigation?^2^
*3.4 Future Plans *– Outline short-term goals (within one year) and long-term goals (two to five years) to further develop your scholarship program.
**4. Supporting Documentation**: eligible activities and products included as appendices:
**Appendix A**
• documents to illustrate your philosophy and goals.
**Appendix B**
• Funding awards, objectives of projects and activities.
• Examples of activities such as teaching interprofessional courses with colleagues from other disciplines, interprofessional curriculum development; seminars, advising students from other disciplines, supervision of an integrated or applied scholarship practicum; scholarship that contributed to the achievement of awards or employment for students. Cross-reference if such evidence is reported in your Scholarship of Teaching Portfolio.
**Appendix C**
• Description of efforts to improve knowledge, skills and methods on scholarship, e.g., seminars, lectures, workshops, and conferences attended. Reflection on your learning.
**Appendix D**
Include details such as names of committees, dates, and the nature of your contribution.
• Activities concerned with scholarship undertaken as a member of a faculty, department, or cross disciplinary committee, sub-committee, *ad hoc *committee, or task force, accreditation committees, program review committees, interdisciplinary curriculum development.
• Faculty resources developed, workshops, conferences organised. Use of your scholarship materials in other faculties, colleges, or universities.
• Participate in orientation sessions for new faculty, seminars, or invited presentations within and outside of the University about your knowledge transfer and exchange activities.
• Invited to consult by scholars in other faculties to improve applied scholarship effectiveness.
**Appendix E**
• Innovative dissemination strategies.
• Peer reviewed documents. Details of books (chapters in books, edited books); articles (refereed, solicited, or non-refereed); papers in conference proceedings (refereed or non-refereed); bibliographies; unpublished professional and technical reports.
• Applied products – innovative programs, policy development, training materials, resource manuals and technical products^21^
• Community dissemination products – newsletters; posters; workshop presentations; community forums, websites, media^21^
**Appendix F**
• Peer reviews from members in the community that your work was meant to benefit.
• Results of evaluations or questionnaires designed by you to obtain feedback about the effectiveness and impact of your activities.
• Solicited and unsolicited letters to attest evidence of your impact and effectiveness.

## Conclusion

It is vital that academic institutions and their scholars are relevant to global communities and change in tenure and promotion systems to support this relevancy are long overdue. Academic institutions have had research, teaching and service in their mandate, but evaluation guidelines, structures and systems have not reflected the multiple forms of scholarship. In this paper, we presented methods for reporting and evaluating two specific dimensions of scholarship, i.e. integration and application. Academic institutions the world over have responsibilities to develop unambiguous interpretations and criteria to evaluate the scholarship of discovery, teaching, integration, and application. Faculty members with enhanced and expanded understandings of scholarship can support academic institutions in their obligations to improve the health and wellbeing in communities. This enlightenment and change could improve the experience of health sciences scholars, create fair merit and promotion processes, strengthen the contributions of academic institutions to society, and legitimize scholarly activities that increase interprofessional collaborations and knowledge exchange and utilization.

## Competing interests

The author(s) declare that they have no competing interests.

## Authors' contributions

AH was responsible for the paper conception and all authors contributed to development. AH drafted the manuscript, MN refined the manuscript, AH, MN and CS made critical revisions to the paper. All authors approved the final version of the paper.

## References

[B1] Mensah LL (1982). Academic espionage: Dysfunctional aspects of the publish or perish ethic. J Adv Nurs.

[B2] Boyer EL (1990). Scholarship Reconsidered: Priorities of the Professoriate.

[B3] Glassick CE, Huber MT, Maeroff GI, Boyer EL (1997). Scholarship Assessed: Evaluation of the Professoriate.

[B4] Day R, Robberecht P, Roed B (1996). Teaching Dossier: A Guide University Teaching Services University of Alberta, Edmonton, Alberta.

[B5] Simpson DE, Marcdante KW, Duthie EH, Sheehan KM, Holloway RL, Towne JB (2000). Valuing educational scholarship at the medical college of Wisconsin. Acad Med.

[B6] Canadian Health Services Research Foundation (2006). The Creative Professional Activity Dossier.

[B7] Canadian Health Services Research Foundation (2006). Develop a Scholarship Framework for Your Field, Faculty or Department.

[B8] Collins BA (1993). A review and integration of knowledge about faculty research productivity. J Prof Nurs.

[B9] Metzler MW (1994). Scholarship reconsidered for the professoriate of 2010. Quest.

[B10] Gros Louis KRR (2000). Evolving the faculty reward system. Acad Med.

[B11] Nowotny H, Scott P, Gibbons M (2001). Re-Thinking Science: Knowledge and the Public in an Age of Uncertainty.

[B12] Johnstone MJ (2007). Journal impact factors: Implications for the nursing profession. Int Nurs Rev.

[B13] Gottlieb LN, Clarke SP (2005). Impact factors and the law of unintended consequences. Can J Nurs Res.

[B14] Hall EO (2001). Scholars and scholarship. Scand J Caring Sci.

[B15] Caelleigh A (2000). Community of scholars. Academic Medicine: Journal of the Association of American Medical Colleges.

[B16] Shapiro ED, Coleman DL (2000). The scholarship of application. Acad Med.

[B17] Marks ES (2000). Defining scholarship at the uniformed services university of the health sciences school of medicine: A study in cultures. Acad Med.

[B18] Glassick CE (2000). Boyer's expanded definitions of scholarship, the standards for assessing scholarship, and the elusiveness of the scholarship of teaching. Acad Med.

[B19] E Fincher R, Work JA (2006). Perspectives on the scholarship of teaching. Med Educ.

[B20] Glassick CE (2000). Reconsidering scholarship. J Public Health Manag Pract.

[B21] Calleson DC, Jordan C, Seifer SD (2005). Community-engaged scholarship: Is faculty work in communities a true academic enterprise?. Acad Med.

[B22] Aday LA, Quill BE (2000). A framework for assessing practice-oriented scholarship in schools of public health. J Public Health Manag Pract.

[B23] Maurana CA, Wolff M, Beck BJ, Simpson DE (2001). Working with our communities: Moving from service to scholarship in the health professions. Educ Health.

[B24] University of Toronto (2006). Academic Promotions Manual Faculty of Medicine, University of Toronto.

[B25] Macfarlane B (2005). The disengaged academic: The retreat from citizenship. Higher Education Quarterly.

[B26] Huber MT (2001). Balancing acts: Designing careers around the scholarship of teaching. Change.

[B27] Institute of Medicine, Committee on Quality of Health Care in America (2001). Crossing the Quality Chasm- A New Health System for the 21st Century.

[B28] D'Amour D, Oandasan I (2004). IECPCP Framework in: Interdisciplinary Education for Collaborative, Patient-Centred Practice: Research and Findings Report.

[B29] Health Canada (2003). Interdisciplinary Education for Collaborative, Patient-Centred Practice Discussion Paper & Research Report Request for Proposal.

[B30] Starck PL (1996). Boyer's multidimensional nature of scholarship: A new framework for schools of nursing. J Prof Nurs.

[B31] Kotter JP (1996). Learning Change.

